# Coherent Feature Extraction with Swarm Intelligence Based Hybrid Adaboost Weighted ELM Classification for Snoring Sound Classification

**DOI:** 10.3390/diagnostics14171857

**Published:** 2024-08-25

**Authors:** Sunil Kumar Prabhakar, Harikumar Rajaguru, Dong-Ok Won

**Affiliations:** 1 Department of Artificial Intelligence Convergence, Chuncheon 24252, Republic of Korea; sunilprabhakar22@gmail.com; 2 Department of ECE, Bannari Amman Institute of Technology, Sathyamangalam 638401, India; harikumarrajaguru@gmail.com

**Keywords:** feature extraction, feature selection, machine learning, classification

## Abstract

For patients suffering from obstructive sleep apnea and sleep-related breathing disorders, snoring is quite common, and it greatly interferes with the quality of life for them and for the people surrounding them. For diagnosing obstructive sleep apnea, snoring is used as a screening parameter, so the exact detection and classification of snoring sounds are quite important. Therefore, automated and very high precision snoring analysis and classification algorithms are required. In this work, initially the features are extracted from six different domains, such as time domain, frequency domain, Discrete Wavelet Transform (DWT) domain, sparse domain, eigen value domain, and cepstral domain. The extracted features are then selected using three efficient feature selection techniques, such as Golden Eagle Optimization (GEO), Salp Swarm Algorithm (SSA), and Refined SSA. The selected features are finally classified with the help of eight traditional machine learning classifiers and two proposed classifiers, such as the Firefly Algorithm-Weighted Extreme Learning Machine hybrid with Adaboost model (FA-WELM-Adaboost) and the Capuchin Search Algorithm-Weighted Extreme Learning Machine hybrid with Adaboost model (CSA-WELM-Adaboost). The analysis is performed on the MPSSC Interspeech dataset, and the best results are obtained when the DWT features with the refined SSA feature selection technique and FA-WELM-Adaboost hybrid classifier are utilized, reporting an Unweighted Average Recall (UAR) of 74.23%. The second-best results are obtained when DWT features are selected with the GEO feature selection technique and a CSA-WELM-Adaboost hybrid classifier is utilized, reporting an UAR of 73.86%.

## 1. Introduction

During sleep, the anatomical structures in the airways vibrate, and this generates a breathing-related event called snoring [1]. The vibration can be on the tonsils, soft palate, or pharyngeal walls. The quality and quantity of sleep are seriously interrupted and disturbed for anyone who shares the neighboring sleep space with the snorer [2]. As a result, daytime sleepiness is induced, enhancing the risk of inattentiveness in workplace and driving scenarios, which might even lead to accidents [3]. If sleep is affected for a chronic period, it can lead to a plethora of problems, such as Coronary Artery Disease (CAD), liver failure, kidney failure, damage to the brain, and damage to other vital organs of the human body. Due to the limited medical resources and negligence of the snoring issue, obstructive sleep apnea could go undiagnosed for many people in the world, thereby leading to more severe complications [4]. If there is no nocturnal respiratory pathology for a snorer, then it will be eliminated to a certain extent by resting on one side instead of the back. To a certain extent, technology has developed, and some gadgets have been proposed to provide notification to snorers during the occurrence of snoring [5]. For most people suffering from obstructive sleep apnea, snoring appears. Various features help to characterize the snoring sounds, such as regularity parameters, spectral parameters, frequency, pitch-related parameters, etc. [6]. The instrument utilized to record the audio signals is cheap, inexpensive, and easy to maintain when compared with Polysomnography (PSG) [7]. To determine the condition of snores, the most vital physiological indicator is the snoring sound signal, and as a result, snoring sound detection and classification research has found a significant place in academic and industry research [8]. Some of the recent and most famous works, including the analysis and classification of snoring sounds, are reviewed as follows.

Based on acoustical analysis of snoring, the respiratory disorders events were classified by Wang et al., who used their own dataset, and when classified with Support Vector Machine (SVM), a recognition rate of 91.14% was obtained [9]. Recurrent Neural Networks (RNNs) were used for snoring sound classification, and the analysis was conducted by Lim et al. on their own dataset, and an accuracy, sensitivity, and F-score of 99.2% were reported [10]. Convolutional RNN with acoustic data segmentation was used by Vesperini et al. for snore detection, where the analysis was performed on an A3-snore dataset and an average precision of 94.92% was obtained [11]. As far as MPSSC dataset is concerned, Mel Cepstral Frequency Coefficients (MFCCs) with ELM and SVM was utilized reporting an UAR of 49.38% [12], Convolutional Neural Networks (CNNs) and AlexNet with VGG19 reported an UAR of 67% [13], Deep CNN reported an UAR of 72.6% [14], SVM reported an UAR of 49.58% [15], Gaussian Mixture Model (GMM) with Deep Neural Networks (DNNs) reported an UAR of 69.71% [16], Local Binary Patterns (LBPs) and Histogram of Oriented Gradients (HOGs) reported an UAR of 66.5% [17], MFCC with SVM reported an UAR of 55.8% [18], CNN with dual convolutions and Gated Recurrent Unit (GRU) reported an UAR of 63.8% [19], wavelet features with classification reported an UAR of 69.4% [20], Conditional Generative Adversarial Networks (GANs) reported an UAR of 67.4% [21] and local dual octal pattern with multilevel DWT hybrid together with Relief and Iterative Neighborhood Component Analysis (RFINCA) and K-Nearest Neighbor (KNN) reported an UAR of 94.65 [22].

The main contributions to the work are as follows.

(a) Once the basic pre-processing is conducted using a simple Independent Component Analysis (ICA) for the snoring sound signals, coherent feature extraction has been implemented, and this is the first time features from almost all the domains have been extracted for the snoring sounds.
(b) The extracted features are selected using three efficient feature selection techniques that make use of meta heuristic techniques.
(c) Finally, it is classified into eight traditional machine learning classifiers and two proposed machine learning classifiers, such as the FA-WELM-Adaboost hybrid model and the CSA-WELM-Adaboost hybrid model.

**Figure 1:**
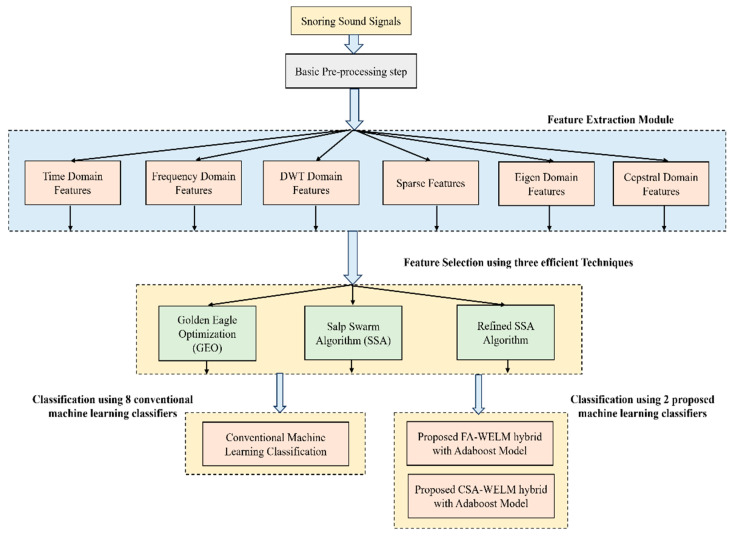
Simplified Illustration of the Work.

Figure 1 shows a simplified illustration of the work.

The organization of the paper is as follows. In Section 2, the coherent feature extraction schemes are discussed in detail, and in Section 3, the efficient feature selection techniques are discussed. Section 4 discusses the implementation of the proposed machine learning classifiers, followed by results and discussion in Section 5 and a conclusion in Section 6.

## 2. Coherent Feature Extraction Schemes

The features extracted are the different time domain features, frequency domain features, DWT domain features, sparse features, eigen domain features, and cepstral domain features.

### 2.1. Time Domain Features

#### 2.1.1. Rhythm-Based Features

A methodical repetitiveness of series or style over time is called rhythm and is traced in environmental sounds, poems, musical instruments, etc. [23]. Some famous rhythm-based features are phoneme duration, speech duration, pulse clarity, articulation rate, beat tracker, pulse metric, beat histogram, band periodicity, etc. Generally, an extended-time band pass auto-correlation for a window of five seconds is used by a measure called the pulse metric, and this feature is utilized in the classification of music genres and the discrimination of music.

#### 2.1.2. Autocorrelation Dependent Features

In the time realm, the self-similarity of a signal is assessed by a measure called autocorrelation. The closeness between the signal and its respective detained class is assessed by this similarity. A strong positive association is indicated by an auto-correlation merit of +1, and a negative association is indicated by an auto-correlation merit of −1, and 0 projects no association. At lag zero, the auto-correlation is projected at value 1, as the signal has a perfect correlation within itself. To assess the periodicity present in the signal, an auto-correlation outcome is utilized. To estimate the pitch of a signal and to scrutinize the musical beats, the auto-correlation function is highly utilized.

#### 2.1.3. Energy-Based Features

As sound signals are non-stationary in nature, their transformation is performed into miniature segments of quasi-stationary signals with the help of the windowing technique. Throughout the signal, the energy present is variable, so predicting a value is not feasible.

Short Time Energy (STE): The computation of STE is conducted here and is expressed as the mean energy per frame. For voiced partitions, the STE is high, and for unvoiced segments, the STE is low. For detecting environmental sounds, music onsets, audio-based server systems, and voiced-unvoiced segments, STE is highly useful.

Temporal centroid:

The time mean over the energy envelope is expressed by this parameter and it is usually utilized in acoustic scene classification and for the recognition of environmental sounds.

Volume:

For a human auditory system, one of the most promising features is loudness, or volume. With a frame, it is expressed as the Root Mean Square (RMS) worth of the magnitude of a signal. It is utilized in acoustic signal classification, speech thresholding, and segmentation, followed by music discrimination.

#### 2.1.4. Amplitude-Based Features

Depending on the temporal envelope of the signal, these features are analyzed.

Amplitude descriptor (AD): Various types of sound envelopes are differentiated by this feature, which is performed by analyzing energy and the duration of signal segments.

Attach, Delay, Sustain, Release (ADSR) envelope: In between musical genres, this ADSR feature is utilized for acoustic signal examination and classification. For most real time sounds, the ADSR envelope feature is not feasible as the decay part and sustain part are not clearly available. So, to tackle this issue, depending on attack and reset mode, a modified envelope is used called the Attach and Release (AR) envelope. Generally, for timbre analysis of music instruments, the ADSR and AR envelopes are utilized.

Shimmer: In a waveform, the pattern-to-pattern discrepancies of the amplitude are computed by Shimmer, and it is used in speaker verification, detection of voice activity, speaker recognition, classification of musical sounds, etc.

Log Attack Time (LAT): It is nothing but the log to the base 10 of the time span between the time initiation and the time it stretches out the steady aspect, and it is widely used for environmental sound recognition and musical onset tracking.

#### 2.1.5. Zero-Crossing Rate (ZCR)

During the acoustic formulation, the appraisal of the sign change of the signal is expressed as a ZCR [24]. The total number of times a signal gradually alters its sign from negative to positive or positive to negative, split by the entire frame period, is expressed by ZCR. In a one second interval, the total number of times the signal crosses the zero extent is expressed by ZCR. The ZCR for the $f^{th}$ frame with the length N is represented as follows:

(1) $$Z(f)=\frac{1}{2N}\sum_{n=1}^{N}\left|\mathrm{sgn}\left[p_{i}\left(n\right)-\mathrm{sgn}\left[p_{i}\left(n-1\right)\right]\right]\right|$$

where $\mathrm{sgn}\left(\cdot \right)$ is nothing but a sign function, i.e.,

(2) $$\mathrm{sgn}\left[p_{i}(N)\right]=\left\{\begin{array}{c} 1,p_{i}(n)\geq 0\\ 0,p_{i}(n)<0 \end{array}\right.$$

To trace the voice activity, an efficient method is ZCR, which helps to ascertain whether a speech is silent, voiced, or unvoiced. For unvoiced positions of the speech, the ZCR is higher. The ZCR is usually zero for silent portions of a clean speech. For predicting the fundamental frequency of a speech, ZCR is highly useful. The important but unintended data about the frequency of the acoustic signal is given by the ZCR, and so this feature is utilized to design the classifier or discriminator very efficiently. An additional kind of ZCR-dependent feature that is quite famous is modified ZCR, as it involves the use of the detrend technique. Equation (1) is slightly modified and specified in Equation (3) as follows:

(3) $$Y(f)=\frac{1}{2N}\sum_{n=1}^{N}\left|\mathrm{sgn}\left[{\hat{z}}_{i}(n)\right]-\mathrm{sgn}\left[{\hat{z}}_{i}(n-1)\right]\right|$$

where, $\hat{z}=\hat{p}-z_{d}$, $\hat{p}$ represents the mean merit of p.

Linear Prediction ZCR: The compact ratio between the ZCR of the actual signal and the ZCR of the predicted error is expressed by this parameter. The linear prediction filter helps in obtaining the prediction error.

### 2.2. Frequency Domain Features

#### 2.2.1. Spectrum Shape Dependent Features

Spectral crest factor: For the sound signal, the peakness of its power spectrum is assessed by the spectral crest factor. To differentiate between tonal and noise-like sounds, this parameter is highly utilized. This parameter is usually low for noise, like acoustics, and very high for tonal sounds.

Spectral flux: It is expressed as a 2-norm of the frame-to-frame spectral amplitude difference vector. The rapid alterations occurring in the frequency energy sound distributions are expressed by spectral flux.

Entropy: The assessment of the regularity of flatness is evaluated by entropy, which can be either Renyi entropy or Shannon’s entropy [25]. In this work, Shannon’s entropy is utilized, and it is computed using the formula, $sum\left(C_{i}\log C_{i}\right)$ where the sample class probabilities are represented by $C_{i}$.

Octave-Based Spectral Contrast (OBSC): The octave scale filters help to assess the sub-bands by computing the variations present between peaks and valleys and are termed OBSC.

Spectral flatness: The consistency in the frequency administration of the power spectrum is assessed by spectral flatness. It is simply a ratio of the geometric mean to the arithmetic mean. This parameter is near zero for harmonic sounds and near one for noise sounds.

Spectral decrease: A good significance is given to the low frequencies as it measures the mean spectral slope of the rate-map specification.

Spectral slope: With the help of linear regression, it is computed, it is worthy, and it is achieved with the assessment of the slope of the proportion of the signal.

Spectral bandwidth: The small bandwidth acoustics are easily assessed from the high frequency acoustics with the help of this second-order statistical value.

Spectral kurtosis: The flatness of the spectrum all over its mean value is expressed by this fourth-order statistical phenomenon called kurtosis. A flat distribution is obtained if the spectral kurtosis value is less than 0. A gaussian distribution is obtained if the spectral kurtosis value is 0. A sharp peak is obtained if the spectral kurtosis value is greater than 0.

Spectral spread: This feature is highly in proximity to the bandwidth of the signal, and it is sometimes called spectral dispersion. Around a particular centroid, the average deviation of the rate-map is expressed by spectral spread. A small spectral spread is found in pure tonal sounds, and a wide spectral spread is found in noise-like signals.

Spectral roll-off: The spectral roll-off location is the specific frequency where a majority of the signal energy is controlled or present beneath this frequency.

Spectral skewness: The spectrum symmetry is measured thoroughly around its arithmetic mean value, and this parameter is a famous third-order statistical value. For voiced parts, spectral skewness is usually high, and for silent segments, it is equal to zero. A symmetric distribution is expressed if the skewness value equals zero. High energy present at the right edge of spectral issuance is indicated if skewness is lower than zero. High energy present to the left edge of spectral issuance is indicated if skewness is much higher than zero.

Spectral center: In the signal spectrum, it is nothing but the assessment of the median frequency. The balancing of lower and higher energies takes place here, as this is a median frequency.

Spectral centroid: The location of the equidistant mass of the spectrum is indicated by the spectral centroid. The brightness of an acoustic signal can be well expressed by this feature. The spectrum is considered here as a dispensation whose values are assigned as frequencies. To analyze the music classification and the timber of music, the spectral centroid is highly useful.

#### 2.2.2. Long-Term Average Spectrum (LTAS)

The unconventional spectral data from the signal is captured by LTAS, which helps classify the pathological speech from the normal speech. The disparity in loudness, nasality, and breath dominance helps to assess the intelligibility of a speech. From every octave filtered speech signal, these cues are captured by LTAS. The assessment of the following parameters is usually performed from the speech signal as follows, range of frame RMS, skewness of frame RMS, kurtosis of frame RMS, assimilated mean frame RMS, normalized range of frame RMS, frame Standard Deviation (STD) normalized by entire band RMS, frame STD normalized by band RMS, and pairwise variability of RMS energy between ensuing frames.

#### 2.2.3. Tonality-Based Features

The sounds of the tonal genre are a very important part of a harmonic acoustic signal. The tonality-dependent audio features are as follows:

Jitter: The variations in the fundamental frequency are computed by jitter. Between the consecutive periods of speech, the average absolute difference is computed by jitter.

Fundamental frequency: In a periodic waveform, the lowest frequency is referred to as the fundamental frequency.

Pitch profile: An exact specification of audio pitch is given by this feature.

Pitch histogram: In its best compact form, the pitch of a signal is explained by the pitch histogram.

Harmonic-to-noise ratio: The compact ratio present between the harmonic aspect of the signal and the remaining aspect of the signal is known by this parameter.

Harmonicity: To differentiate between noise-like sounds and tonal sounds, this feature is used. In the time or frequency domain, auto-correlation justification is used so that periodicity is found in sound.

#### 2.2.4. Peak Frequency

The frequency of a maximum power is expressed by its peak frequency. The most influential frequency component present in the signal is estimated by peak frequency. The rudimentary frequency of the signal is also computed with the help of the peak frequency.

#### 2.2.5. Autoregression-Based Features

The extraction of auto-regression-dependent features is conducted through linear prediction and scrutiny of a signal [26]. The habitual auto-regression-dependent features include linear spectral frequency, Linear Predictive Coding (LPC) coefficients, and Code Excited Linear Prediction (CELP).

Linear Spectral Frequency: It is generally utilized in speech coding, and it is also termed linear spectral pairs. The linear prediction coefficients are specified by LSF so that they can be transmitted over the channel. The average of both anti palindromic and palindromic polynomials is specified as a linear prediction polynomial. The vocal track is specified when the glottis closes with the palindromic polynomial, and the vocal track is represented when the glottis opens with the anti-palindromic polynomial. The identification of the rotos is simply obtained from the LSF specifications of the linear prediction polynomial.

LPC coefficients: The redundancy in a signal is eliminated by LPC. By hybridizing the previously known coefficients, the prediction of the next values is performed in a linear manner. The spectral envelope of a digital speech is specified by the LPC, and hence it is used for audio retrieval and audio segmentation purposes.

CELP: It is highly dependent on the techniques as follows. The vocal trust is minimized so that the linear prediction model is utilized. In the linear prediction model, fixed adaptive code book entries are utilized as the excitation signal. Only in a closed loop is the search performed exhaustively, and it is achieved in a weighted domain. A better quality is provided by the CELP speech coding algorithm.

#### 2.2.6. STFT-Based Features

A signal with time on one specific axis and frequency on another specific axis is projected by a time–frequency transform with the aid of a time–frequency transform [27]. With the aid of time–frequency distribution, the time–frequency investigation could be acquired. Over the interval, the contrast in signal amplitude is shown by the time-domain, and the frequency data is given by the magnitude of the frequency realm in the frequency domain. The most common way to have a time–frequency characterization is by using STFT. For the analysis of non-stationary parts of a signal, such as drifts, time–frequency representation features are highly effective.

Spectrum envelope: To generate or model a mitigated spectrogram of an acoustic signal, the spectrum envelope is used as it is expressed as a log-frequency power spectrum of a particular signal. When the generation of the spectral envelope is conducted by the linear prediction technique, it is termed the linear prediction spectral envelope.

Sub-band energy ratio computation: With the help of STFT and Fast Fourier Transform (FFT), the signal is broken into various frequency bands by sub-band coding, and then, in an independent manner, encoding is performed individually. Along the various frequency bands, it can be expressed as an estimation of normalized signal energy.

Time–frequency matrix: The conversion of a time-domain signal into a time–frequency specification can be performed with the help of STFT. Different decomposition schemes are utilized to mitigate the dimension of the time–frequency matrix, and some of them are linear time–frequency matrix, quadratic time–frequency matrix, positive time–frequency matrix, matching pursuit, etc. In this work, matching pursuit is used as it utilizes a non-orthogonal basis function so that a signal could be decomposed into Gabor atoms. This enables the possibility of good scaling and modulation.

Group Delay Function (GDF): The phase information is usually avoided when we deal with the frequency domain examination of an acoustic signal, as the concentration is performed only on real values. By computing the derivative of this phase, the information in the STFT phase function is performed, and it is termed the group delay function. The vital data about the temporal in a signal is expressed by GDF, and it can be maximum phase/minimum phase/or mixed phase GDF.

Stereo Panning Spectrum Feature (SPSF): The conversion of stereo audio into mono channel audio is usually conducted through audio signal processing. However, the data available because of stereo panning is not used entirely, so to avoid this problem, the stereo panning spectrum is used. Depending on the cross-channel metric, the frequency-domain source recognition method is termed the panning index. The signal is generally held between −1 and +1 in the stereo panning spectrum. Different statistical features can be obtained from the stereo panning spectrum, such as panning RMS for a specific frequency band, panning index derivatives, high–frequency panning, and low–frequency panning.

### 2.3. DWT Domain Features

A famous technique to reconstruct the time-realm acoustic signal into a time–frequency specification is by using a wavelet transform [28]. The inner outcome of the signal is computed with a particular member from wavelet descent. DWT and Continuous Wavelet Transform (CWT) are two different varieties of wavelets. Extracting information from audio signals can be easily conducted by DWT. The shortcomings of the STFT are easily overcome by it, so that uniform time–frequency resolution is provided. A high time resolution with low–frequency resolution is obtained for higher frequencies by DWT, and a high–frequency resolution with low time resolution is obtained for lower frequencies by DWT. The wavelet transform helps generate the approximations and detailed coefficients so that a good understanding of the signal is obtained. Such detailed coefficients, represented as approximations, are termed wavelet features. The wavelet packet decomposition, or wavelet transform, is used to extract the wavelet features. The decomposition of the approximation coefficients is conducted by wavelets, while both the approximation and detailed components are broken down in wavelet packet decomposition. The conventional features or coefficients extracted from them are used directly as wavelet features.

### 2.4. Sparse Domain Features

Very few elements are non-zero, and most of the elements are zero in a sparse matrix. This concept of sparse features is applied to acoustic signals, which have only a small amount of non-zero ingredients [29]. In the frequency realm, one single spike is used to indicate a pure tonal signal, and so it could be implemented that a signal is quite sparse in the frequency realm. Thus, if sparsity is achieved, only a few features are necessary to indicate a signal. Through various domains such as wavelet domain, time–frequency domain, frequency domain, time domain, and cepstral domain, sparsity could be achieved. The extraction of realm-specific features can be conducted easily if sparsity is achieved. The extraction of cepstral features can be conducted from the sparse nature of the signal if the sparsity is there in the cepstral domain. The sparsity can be achieved by basic pursuit, matching pursuit, Orthogonal Matching Pursuit (OMP), coordinate descent, state wise greedy technique, etc.

### 2.5. Eigen Domain Features

The extraction of features from the eigen vectors of an acoustic signal is called eigen domain features [30]. The dominant vector assessed in the signal is the eigen vector of an acoustic signal. With the help of different techniques, the dominant vector is obtained. The techniques can be Singular Value Decomposition (SVD), Principal Component Analysis (PCA), Independent Component Analysis (ICA) etc. The actual acoustic signal is projected into the eigen-vector spaces with the aid of these techniques.

### 2.6. Cepstral Domain Features

By computing the inverse Fourier transform of the logarithm of the signal spectrum, a cepstrum is obtained [31]. Cepstrums can be real, complex, power, or phase cepstrums, however, for audio signal processing, power cepstrums are highly useful. When the cepstrum is analyzed comprehensively, it is termed cepstrum analysis. Some of the cepstral domain features are discussed as follows:

Linear Prediction Cepstral Coefficients (LPCCs):

A huge number of merits are possessed by the cepstrum, such as compactness, source-filter separation, source-filter separation, orthogonality, etc. The cepstrum coefficients are quite robust with these properties, and hence they are suitable for machine learning and deep learning. The Linear Prediction Coefficients (LPCs) should be altered to the cepstral realm as they are too sentient to numerical position. The obtained transformed coefficients are termed LPCCs.

Mel Frequency Cepstral Coefficients (MFCCs):

From the cepstral specification of an acoustic clip, the MFCCs are derived. The short-time power spectrum of an acoustic clip is specified by MFCC, and it is highly dependent on the Discrete Cosine Transform (DCT) of the log power spectrum on a nonlinear mel scale. There is an equal spacing of frequency bands on the mel-scale where the human hearing arrangement is mimicked quite closely.

Preceptual Linear Prediction (PLP) cepstral coefficients:

It is dependent on three important ideas, such as the intensity loudness power law, the critical band spectral revolution, and the equal-loudness curve. Before auto-regressive modeling, preceptual processing is performed, and the derivation of the PLP coefficients is obtained from the LPC. The conversion of the linear coefficients to cepstral coefficients is conducted once this processing is performed.

Greenwood Function Cepstral Coefficients (GFCCs):

As an overall form of MFCCs, GFCCs were introduced. Mel-scale features are utilized by GFCCs so that a good vocal representation is achieved. With the help of the Greenwood equation, the least and highest frequency ranges are ascertained [32].

Relative Spectral PLP (RASTA-PLP) features:

For the energy in every frequency sub-band, a band pass filter is implemented so that short-term noise variations are smoothed and the constant offsets are removed. The noise cancellation feature is incorporated here in RASTA-PLP.

Gammastone Cepstral Coefficients (GTCCs):

Good noise robustness is provided by GTCCs in the field of automatic speech recognition. They are highly dependent on gammatone filter banks, so an exact time–frequency specification of a sound signal can be obtained [33]. The extrication procedures of both MFCCs and GTCCs are similar to each other, as MFCCs use mel-filter banks and GTTCs use gammatone filter banks. The primary and secondary order derivatives of GTCCs are projected as subsidiary features like delta–delta GTCCs, delta GTCCs, etc.

## 3. Efficient Feature Selection Techniques

The efficient feature selection techniques chosen in his paper are GEO, SSA, and refined SSA.

### 3.1. GEO Dependent Techniques for Feature Selection

The behavior of golden eagles is simulated by the GEO algorithm, which includes choosing prey, attacking forces, hunting prey cruising, and force adjustment under various stages [34]. When comparing other algorithms, the resulting mathematical model of GEO provides good superiority, so it is used for feature selection in our work [35].

#### 3.1.1. GEO Model

The spiral movement of the golden eagles is simulated during the preying process when the simulation of GEO happens. A feeding path is planned by every golden eagle in the natural environment when a targeted prey is found. A good capacity cannot be guaranteed by the golden eagles to replenish themselves if there is a direct attack on prey without any arrangement. The endless cruise always progresses to more consumption, on the other hand. To obtain the best hunting, these two forces must be balanced by the golden eagles. In the early stage, more inclination is required, and in the later stage, more aggressive attacks on the prey are conducted. The location of prey with other prey is shared by the golden eagles so that the space near the prey is searched thoroughly, thereby making the entire population better at prediction. In the population, every individual has their own memory when the mathematical replica of GEO is developed. The population size always equals the number of memories, and the population size is expressed as $P_{size}$ which denotes the entire number of individuals in the population. The versatile position is remembered by every golden eagle’s memory, which it traced when the search process happened. The memory is updated by the golden eagle when a better position is found. The best position in the memory of other golden eagle h is considered by the golden eagle g at every iteration i. With the help of one-to-one random mapping, the predation target is achieved here. Depending on the targeted prey positions, the attack and cruise vectors are computed sequentially. For the next iteration, the new position is assessed so that the movement vector of golden eagle g is attained. Once the predation target is chosen, the attack vector is computed by the golden eagle g as follows:

(4) $${\vec{V}}_{g}={{\vec{T}}_{h}}^{*}-{\vec{T}}_{g}$$

where the attack vector of golden eagle g is represented by ${\vec{V}}_{g}$, the target prey vector of eagle g and the best position vector of eagle h is represented by ${{\vec{T}}_{h}}^{*}$, and the present location of golden eagle g is represented by ${\vec{T}}_{g}$. The distance and direction are emphasized by the attack vector, which corresponds to the prey. It always inclines to progress towards better solutions, so the exploitation capacity of the algorithm is easily reflected. Once the attack vector is obtained, the golden eagle g is computed by the cruise vector. The cruise vector is a simple random vector situated in the hyperplane at right angles to the attack vector. A spatial plane s−1 composes the hyperplane that could easily split the s —dimensional space. The cruise vector is considered as ${\vec{W}}_{g}$ in the hyperplane while considering ${\vec{V}}_{g}$ as a vertical vector. By analyzing the hyperplane properties, the following equation is obtained as follows:

(5) $$\sum_{k=1}^{N}v_{g}^{k}w_{g}^{k}=0$$

where the constituent of the attack vector ${\vec{V}}_{i}$ in the $k^{th}$ dimension is represented as $v_{g}^{k},k\in \left[1,2,....,S\right]$. $w_{g}^{k}$ represents the elements of the cruise vector ${\vec{W}}_{g}$. Random values can be generated by the cruise vector in s−1 dimensions instead of s dimensions. To make sure that perpendicularity exists on the normal vector, the $q^{th}$ dimension of the random vector of the hyperplane should be standardized. The generation of the cruise vector is performed as follows. The random choosing of the $q^{th}$ dimension is conducted as the fixed dimension. In the range of [−1,1], the s−1 values other than the $q^{th}$ dimension are considered arbitrarily. With the help of the reverse derivation of Equation (5), the $q^{th}$ dimension could be easily obtained. The $k^{th}$ dimension constituent of the cruising vector ${\vec{W}}_{g}$, i.e., $w_{g}^{k}$ is expressed by Equation (6) as follows:

(6) $$w_{g}^{k}=\left\{\begin{array}{c} 2r_{w}-1\;if\;k\neq q\\ \frac{-\sum_{k=1;k\neq q}^{N}v_{g}^{k}w_{g}^{k}}{v_{g}^{q}}\;else \end{array}\right.$$

where the random value $r_{w}$ is placed between [0,1]. While preying, for the next migration, a sub-vector exists, and the golden eagle is enabled by the cruising vector so that the search is taken into account beyond the prey range and so the exploration capacity is highlighted. With the computation of attack and cruise vectors, the step vectors in iteration t are obtained by the golden eagle g with the help of two sub-vector compositions and expressed as follows.

(7) $$\Delta t_{i}={\vec{r}}_{1}c_{v}\frac{{\vec{V}}_{g}}{\left\|{\vec{V}}_{g}\right\|}+{\vec{r}}_{2}c_{w}\frac{{\vec{W}}_{g}}{\left\|{\vec{W}}_{g}\right\|}$$

where the random vectors are denoted as ${\vec{r}}_{1}$ and ${\vec{r}}_{2}$ with elements [0,1]. $\left\|{\vec{V}}_{g}\right\|$ and $\left\|{\vec{W}}_{g}\right\|$ represent the Euclidean norm of the attack and cruise vector expressed in Equation (8) as follows:

(8) $$\left\|{\vec{V}}_{g}\right\|=\sqrt{\Sigma_{k=1}^{N}v_{k}^{2}},\left\|{\vec{W}}_{g}\right\|=\sqrt{\sum_{k=1}^{N}w_{k}^{2}}$$

where the attack and cruise weight coefficients are represented as $c_{v}$ and $c_{w}$, respectively, which usually change with iteration in a linear manner as shown in Equation (9).

(9) $$\left\{\begin{array}{c} c_{v}=c_{v}^{0}+\frac{i}{I}\left\|c_{v}^{I}-c_{v}^{O}\right\|\\ c_{w}=c_{w}^{0}+\frac{i}{I}\left\|c_{w}^{I}-c_{w}^{O}\right\| \end{array}\right.$$

where the current iteration is indicated by i and the highest iteration is specified by I. $c_{v}^{O}$ and $c_{v}^{I}$ represent the start and end values of the coefficients of attack weight $c_{v}$. The start and end values of the cruise weight coefficient $c_{w}$ are represented by $c_{w}^{O}$ and $c_{w}^{I}$. The $c_{v}$ and $c_{w}$ are usually correlated in a positive and negative manner with each other. The value of $c_{v}$ is small and the value of $c_{w}$ is large at the start of the iteration. It is ensured that more regions are explored by the golden eagle in the population. As iteration increases, $c_{v}$ increases and $c_{w}$ decreases so that the tendency of golden eagles increases, leading to the exploitation stage from the exploration stage. Thus, when the iteration ends, more exploitation is performed by golden eagles so that better solutions are obtained in a short span of time. To get good support between exploration and exploitation in this algorithm, the dynamic shift of coefficients is greatly enabled. For the golden eagle g, the new position in iteration i+1 is attained by adding the present position in iteration i with the step vector as expressed below.

(10) $$t_{g}^{k}(i+1)=t_{g}^{k}(i)+\Delta t_{g}^{k}$$

Fitness is reevaluated by every golden eagle after it moves to the new position so that it can compare itself with the best position in memory. The novel position can easily replace the memory value if the novel position is better. Unless the termination condition is met, this process is repeated in every iteration. The GEO algorithm is explained in Algorithm 1.
**Algorithm 1:** GEO Algorithm$\mathrm{Assign}\;\mathrm{Population}\;\mathrm{size}\;P_{size},$ the maximum generation I $\mathrm{Assign}\;\mathrm{start}\;\mathrm{and}\;\mathrm{end}\;\mathrm{values}\;\mathrm{of}\;\mathrm{the}\;\mathrm{attack}\;\mathrm{and}\;\mathrm{cruise}\;\mathrm{weighting}\;\mathrm{coefficients}\;c_{v}^{0},c_{v}^{T},c_{w}^{0}$ $\mathrm{and}\;c_{w}^{T}$ Initialize Random population Assign present position of golden eagles as memory M Compute fitness based on the position of every golden eagle. Assign the current cycle counter i=1 while i<=I do $\mathrm{for}\;g=1\begin{array}{cc} to\begin{array}{cc} P_{size} & do \end{array} & \end{array}$ Random selection of prey from memory population Compute attack vector from (4) if length of attack vector ≠0, then Compute cruise vector based on Equation (6) Compute step vector based on Equation (7) Update position by Equation (10) Compute fitness for the new position $t_{g}^{i+1}$ $\mathrm{if}\;f\left(t_{g}^{i+1}\right)<f\left(M_{g}^{i}\right)$, then
$M_{g}^{i+1}=t_{g}^{i+1}$ i=i+1 Output the best solution of golden eagle.

#### 3.1.2. Feature Selection Using Binary GEO

The solutions must be generally discrete in form, so a binary form of GEO is utilized. The origin of these solutions can be made intermittent and feasible with binary GEO. A 1D vector of length is used here to define the solution of every search agent so that it can match the entire number of features in the set. Only values of 0 or 1 could be considered by elements of the vector, proving that the features could either be discarded or chosen wisely. A rational transform technique is used to express the solution specification. In every iteration, the generation of continuous results is conducted, and they are converted into feasible solutions. By replacing Equation (10) with Equation (11), the continuous to discrete form could be as follows:

(11) $$t_{g}^{k}(i+1)=\left\{\begin{array}{c} 1\;if\;t_{g}^{k}(i)+\Delta t_{g}^{k}\leq \theta \\ 0\;else \end{array}\right.$$

where the value of $\Delta t_{g}$ in the $k^{th}$ dimension is expressed as $\Delta t_{g}^{k}$. The threshold value is considered here as θ and is chosen here as the hyperparameter. To determine the quality of novel generated solutions, it is necessary to pick an evaluation metric. If BGEO is considered a wrapper approach, then it has two main objectives, such as feature subset minimization and classification accuracy maximization. The addition or removal of a feature does not matter here, as it does not affect the performance of classification accuracy as it is not mutually exclusive. The fitness function is utilized here so that the balance between the two objective functions can be achieved as follows:

(12) $$f\left(p\right)=\alpha \times \gamma_{p}(D)+\beta \times \frac{\left|p\right|}{N}$$

The evaluated subset of features is expressed as p and the classification error rate of the classifier is specified as $\gamma_{p}(D)$. The number of features within p is specified as $\left|p\right|$ and the entire number of features in the dataset is expressed as N. The significant parameters are α and β, we represent it as $\alpha \in \left[0,1\right]$ and β=1−α. The significance of the classification error rate and the total number of chosen features are specified by α and β, respectively.

### 3.2. Salp Swarm Algorithm (SSA)

**Figure 2:**
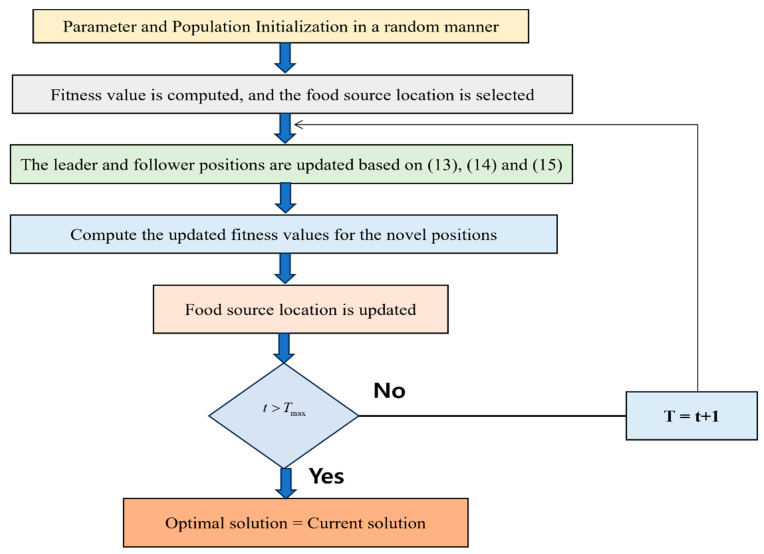
Simplified Illustration of the SSA.

The *salp* is a type of miniature glial chordate animal that survives in the deep sea. A good connection between many individuals takes place so that long chained colonies are formed when the asexual period happens. Depending on the aggregate behavior of the *salps*, this algorithm was developed [36]. The *salp* population is split into two distinct groups in this algorithm. At the top of the *salp* swarm chain, an individual is present and is projected as a leader, and the followers are nothing but the remaining individuals in the group. In an N-dimensional search space, the continuous updating of the individual positions of the *salp* swarm happens, thereby allowing the food source location to be easily traced. With the help of the rule in Equation (13), the position of the leader in the population of *salps* is updated as follows:

(13) $$d_{k}^{1}=\left\{\begin{array}{c} H_{k}+r_{1}\left(\left(up_{k}-lp_{k}\right)r_{2}+lp_{k}\right),r_{3}\geq 0\\ H_{k}-r_{1}\left(\left(up_{k}-lp_{k}\right)r_{2}+lp_{k}\right),r_{3}<0 \end{array}\right.$$

where the leader position in the $k^{th}$ dimensional *salp* chain is indicated as $d_{k}^{1}$. The food source position in the $k^{th}$ dimension is represented as $H_{k}$. In the $k^{th}$ dimension, the upper and lower bounds are indicated as $up_{k}$ and $lp_{k}$, respectively. In the interval [0,1], the random numbers are indicated as $r_{2}$ and $r_{3}$. In the algorithm iteration, a major adaptive factor is represented by $r_{1}$ and is represented as follows:

(14) $$r_{1}=2e^{-{\left(\frac{4i}{I_{\max}}\right)}^{2}}$$

where the maximum number of iterations is specified as $I_{\max}$ and the current number of iterations is indicated as i. When the updation of the leader’s position is achieved, the position of the followers’ changes and is expressed as:

(15) $$d_{k}^{i^{’}}=\frac{1}{2}\left(d_{k}^{i}+d_{k}^{i-1}\right)$$

where the position of the $i^{th}$ follower in the $k^{th}$ dimension is represented as $d_{i}^{k}$ before the update. The position of the $i^{th}$ follower in the $k^{th}$ dimension is represented as $d_{k}^{i^{’}}$ after the update. Figure 2 shows the flowchart for the SSA.

#### 3.2.1. Refined *Salp* Swarm Algorithm

The slow convergence of the *salp* swarm and the issues of local optimization are solved by the refined SSA [37]. To generate the start conditions of iterations, the introduction of tent chaotic mappings was conducted so that the improvement in each stage of iteration is justified and the convergence speed is improved. The local and global exploration capability of SSA is improved by implementing a step adjustment technique for inertia weights. With the aid of a simulated annealing policy, the local optimal solutions were escaped at the final stages of iterations.

(A) Generation of Tent Chaotic mapping:

The initialization of the *salp* population was performed at the start, which was generated by a chaotic tent map and expressed as:

(16) $$d_{t+1}^{i}=\left\{\begin{array}{c} 2d_{t}^{i},0\leq d_{t}\leq \frac{1}{2}\\ 2\left(1-d_{t}^{i}\right),\frac{1}{2}\leq d_{t}^{i}\leq 1 \end{array}\right.$$

**Figure 3:**
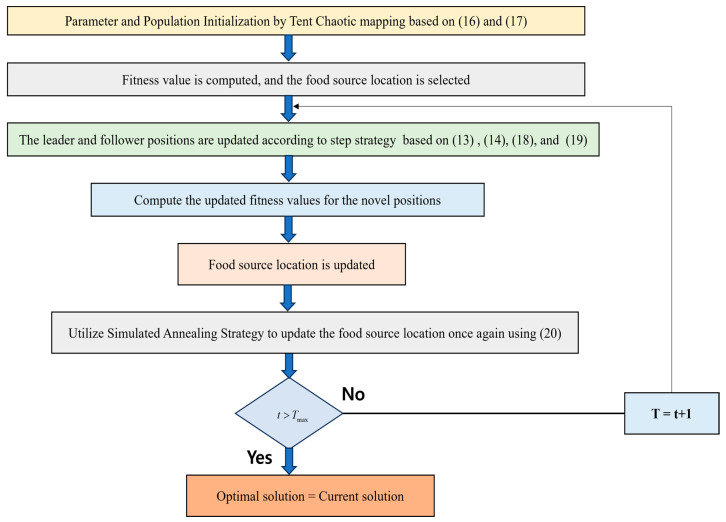
Simplified Illustration of refined SSA.

The above Equation (16) can be transformed to the following using the Bernoulli shift transformation as follows:

(17) $$d_{t+1}^{i}=\left(2d_{t}^{i}\right)\mathrm{mod}1$$

(B) Insertion of stepped inertia weights:

The concept of stepped inertia weights can be represented as follows:

(18) $$d_{k}^{i^{’}}=\frac{1}{2}\left(d_{k}^{i}+w\left(l\right)d_{k}^{i-1}\right)$$

(19) $$w\left(l\right)=\left\{\begin{array}{c} w_{\max},t/t_{\max}\leq \lambda \\ w_{\min},t/t_{\max}>\lambda \end{array}\right.$$

where the minimum inertia weight is specified as $w_{\min}$ and the maximum inertia weight is specified as $w_{\max}$.

(C) Simulated Annealing algorithm:

With the help of a simulated annealing algorithm, the updated food source locations are adjusted so that the poor-quality groups are accepted as follows:

(20) $$S=\left\{\begin{array}{c} 1,f\left(D^{i^{’}}\right)<f\left(D^{i}\right)\\ \exp\left(-\left(f\left(D^{i^{;}}\right)-f\left(D^{i}\right)\right)/T_{t}\right),f\left(D^{i^{’}}\right)\geq f\left(D^{i}\right) \end{array}\right.$$

where the fitness value of the $i^{th}$ individual in the *salp* population is represented as $f\left(D^{i^{’}}\right)$. The $t^{th}$ iteration temperature is indicated by $T_{t}$. Figure 3 shows the flowchart for refined SSA.

#### 3.2.2. Feature Selection by SSA

The candidate inputs of the classifiers are nothing but the vector elements. When utilizing swarm intelligence for feature selection problems, a real vector is projected as the set of individuals in one population, with every element normalized in the specific range of [0,1]. By means of real vector discretization, the features are selected and evaluated for binary values in the range of {0,1}.

(21) $$d_{dk}^{i}(t)=\left\{\begin{array}{l} 1,d_{k}^{i}(t)>0.5\\ 0,else \end{array}\right.$$

where the continuous value position of the $k^{th}$ dimension for the $i^{th}$ individual is represented as $d_{k}^{i}(t)$. The discrete binary value of $d_{k}^{i}(t)$ is represented by $d_{dk}^{i}(t)$. If a value of “0” arises, then it implies that the $k^{th}$ feature was not chosen. If a value of “1” arises, it implies that the $k^{th}$ feature was chosen. For further prediction testing, the chosen subset of the entire feature set is fed to the classifiers.

## 4. ELM, Weighted Version of ELM and the Concept of Weighted Kernel ELM (WKELM)

A famous single-hidden layer feedforward neural network is ELM [38]. The weight linking vector v and bias b are the hidden neuron parameters, and the assignment of them is performed in a random manner. With the help of Moore–Penrose generalization, the assessment of the output weights β can be performed. The N number of samples is assumed to be in the training set $P=\left\{\left.\left(p_{i},q_{i}\right)\right|i=1,2,\;\ldots ,\;N,p_{i}\in {ℜ}^{d},q_{i}\in \left\{1,2,\;\ldots ,\;C\right\}\right\}$ and D number of nodes are present in the hidden layer. The feature dimension number is specified by d and the total number of classes is specified by C. The Original ELM (OLEM) output can be specified as:

(22) $$\sum_{k=1}^{F}\beta_{k}s\left(\langle v_{k},p_{i}\rangle +b_{k}\right)=T_{i},i=1,2,\;\ldots ,\;N$$

If the sample $p_{i}$ belongs to this class, then $T_{i}$ is represented as 1; otherwise, it is represented as −1. The activation function is specified as s and the inner product of the vectors is specified by $\langle \cdot ,\cdot \rangle$. With the help of a matrix model, the equation can be represented as follows:

(23) Hβ=T

With the minimal norm, the least squares solution is obtained as a regularization term as specified as follows:

(24) $$\beta =H^{†}T=\left\{\begin{array}{c} H^{T}{\left(\lambda I+HH^{T}\right)}^{-1}T\;when\;N\leq D\\ {\left(\lambda I+H^{T}H\right)}^{-1}H^{T}T\;when\;N\geq D \end{array}\right.$$

The Moore–Penrose generalized inverse is expressed by t. For a normal sample p, the actual ELM classifier prediction label is expressed as:

(25) $$label\left(p\right)=\underset{i}{\arg\max}\;f_{i}(p),i=1,2,\;\ldots ,\;C$$

where,

(26) $$f\left(p\right)=\left[f_{1}(p),f_{2}(p),\;\ldots ,\;f_{c}(p)\right]$$

and

(27) $$f(p)=\left\{\begin{array}{c} h(p)H^{T}{\left(\lambda I+HH^{T}\right)}^{-1}T\;when\;N\leq D\\ h(p){\left(\lambda I+H^{T}H\right)}^{-1}H^{T}T\;when\;N\geq D \end{array}\right.$$

In the training set, the weight of every sample is treated equally.

The weight of $p_{i}$ should be very large if $p_{i}$ arrives from a minority class, so that the classification accuracy is improved. If the weighted least squares solution is the N×N diagonal matrix U is related to the weight w of the training sample $p_{i}$. The Equation (24) can be revised as follows in WELM and expressed as follows:

(28) $$\beta =H^{†}T=\left\{\begin{array}{c} H^{T}{\left(\lambda I+UHH^{T}\right)}^{-1}UT\;when\;N\leq D\\ {\left(\lambda I+H^{T}UH\right)}^{-1}UH^{T}T\;when\;N\geq D \end{array}\right.$$

WELM techniques can easily adopt the kernel method, as they are inspired by kernel techniques in SVMs. The kernel matrix can be expressed as follows:

(29) $$\Omega_{ELM}=HH^{T},\Omega_{ELM_{z,t}}=h\left(p_{z}\right)h\left(p_{t}\right)=K\left(p_{z},p_{t}\right)$$

In the training set, the number of samples is regarded as equal to the total number of hidden layers D in the Weighted Kernel ELM (WKELM). Therefore when N≤D, the Equations (27) and (28) can be revised and expressed as follows:

(30) $$\beta =H^{†}T=H^{T}{\left(\lambda I+U\Omega \right)}^{-1}WT$$

(31) $$f(p)=\left[k\left(p,p_{1}\right),....,k\left(p,p_{N}\right)\right]{\left[\lambda I+U\Omega \right]}^{-1}UT$$

### 4.1. Composited Kernel ELM (CKELM)

The spectral and contextual information can be easily hybridized by the composited kernels, and it has good versatility. The kernel function could be adopted as a weighted summation kernel for any hybrid classifier [39]. The spectral and spatial content are balanced by this kernel technique as follows:

(32) $$k\left(p_{i},p_{j}\right)=\mu K_{s}\left(p_{i}^{s},p_{j}^{s}\right)+\left(1-\mu \right)K_{w}\left(p_{i}^{w},p_{j}^{w}\right)$$

The vectors extricated from the spatial feature are represented as $p^{s}$ and the vectors extricated from the spectral band are represented as $p^{w}$. In between the spectral and spatial kernel techniques, the balance coefficient is represented by μ and is in the range of [0.1]. The mean and standard deviation are implemented in a local $\left(2w_{r}+1\right)\times \left(2w_{r}+1\right)$ window per spectral band, where the window radius is denoted as $w_{r}$. For the spatial kernel, the RBF is implemented as follows:

(33) $$K_{s}\left(p_{i}^{s},p_{j}^{s}\right)=\exp\left(-\gamma {\left\|p_{i}^{s}-p_{j}^{s}\right\|}^{2}\right),\gamma \in {ℜ}^{+}$$

For the spectral kernel, the polynomial function is implemented as follows:

(34) $$K_{w}\left(p_{i}^{w},p_{j}^{w}\right)={\left(\langle p_{i}^{w},p_{j}^{w}\rangle +1\right)}^{d},d\in Z^{+}$$

### 4.2. Hybrid Adaboost with Weighted ELM

In an adaptive manner, a single strong classifier is generated by hybridizing a lot of weak classifiers using the Adaboost algorithm [40]. In this algorithm, the training procedure is a serial iteration. With the help of kernel functions, which are user-predefined, the transformation of every sample is conducted into a new feature vector before every iteration. Depending on the classifier’s performance, the weight adjustment of every sample is performed. The sample weight would be enhanced if a particular sample was misclassified by a prior classifier. Therefore, in this iteration, a vital role is played by the sample, so the classifier is forced to concentrate and manage the misclassified samples. In the training set, the initial weight for all samples is set by the following:

(35) $$w_{ii}^{o}\left(p_{i}\right)=\frac{1}{c\neq m_{i}}$$

where the number of samples that belong to the class $m_{i}$ is denoted as $\neq m_{i}$. Imbalance can happen sometimes, so the actual Adaboost algorithm using equal weights for each sample is not implemented. In each class, depending on the imbalanced number of samples, the sample weights are adjusted. In the same procedure, the summation of weights per class is also conducted. Then, for the Adaboost framework, M iterations are initiated. By using weighted CKELM, the classifier is trained and constructed at every iteration. The computation of the classifier accuracy is performed, and the training set sample weights are updated as follows:

(36) $$c_{i}w_{ii}^{m+1}=\frac{w_{ii}^{m}\exp\left(-\alpha_{t}I\left(label^{m}\left(p_{i}\right),q_{i}\right)\right)}{Z_{q_{i}}^{t}}$$

where the classifier weight is indicated as $\alpha_{t}$ and is expressed as follows:

(37) $$\alpha_{t}=\ln\left(\left(1-\varepsilon_{t}\right)/\varepsilon_{t}\right)+\ln\left(C-1\right)$$

The error rate of this classifier is denoted by $\varepsilon_{t}$ and is expressed as follows:

(38) $$\varepsilon_{t}=\sum_{i}w_{ii}^{m}\left(I-\left(label^{m}\left(p_{i}\right),q_{i}\right)\right)$$

The indicator function is represented by $I\left(\cdot ,\cdot \right)$.

(39) $$I\left(v,b\right)=\left\{\begin{array}{c} 1\;when\;v=b\\ 0\;when\;v\neq b \end{array}\right.$$

and $Z_{q_{i}}^{t}$ denotes a normalization denominator.

(40) $$\Sigma_{q_{i}=k}w_{ii}^{m+1}(p_{i})=\frac{1}{c}$$

In the training process, every class plays a vital role so that the classifiers do not have an inclination towards the class, which would have a huge number of samples. Based on the weight voted concept, the Adaboost KELM determines the label in the test process if a new sample is considered, and it is expressed as follows:

(41) $$label(p)=\underset{i}{\arg\max}\sum_{t=1}^{T}\alpha_{t}I\left(label^{t}(p),i\right),i=1,2,\;\ldots ,\;C$$

The hybrid Adaboost with weighted ELM is much better than the WELM concept. To rebalance the significance of every sample, weights are introduced in the framework. For the imbalanced datasets, the classification accuracy must be improved, and so weights are highly useful for that purpose. The adaptive adjustment of the sample weights is managed efficiently based on the present error rate of the classifier and the training sample imbalance. If the incorrect classification of the sample happens, then the weight of the training sample is made larger. To rebalance the significance of every class, the weights are rearranged based on every training sample. If a new sample arrives, the Adaboost framework generates the outputs from a collection of classifiers, which are then hybridized together to form a final decision, making it more efficient and robust. At every iteration, the kernel matrix of the training set is attained, and so the hybrid Adaboost with WELM classifier seems to be a very efficient classifier.

### 4.3. Proposed Application of Firefly Algorithm to Optimize Weighted ELM Model

In earlier literature, researchers have provided or implemented the results with ELM and Hybrid Adaboost-ELM. Some researchers have also tried to implement the use of swarm intelligence with Adaboost-ELM classifiers. So, in this paper, the authors tried to bring some novelty by using FA [41] and a Weighted ELM classifier hybrid with the Adaboost classifier. Then again, we replaced FA with CSA [42] and tried the analysis with weighted ELM classifiers hybridized with Adaboost classifiers. Based on the swarm intelligence concept, FA was developed, and it serves as an important stochastic search method. The fireflies progressing towards the brightest fireflies than the brighter one passes the way for the origin of this algorithm [41]. The answer to the optimization issue is specified by the firefly position in the search space. The adaptation value is highly related to the brightness of this optimization issue. Unless it reaches the termination condition, the firefly progresses towards the brighter firefly, and then the optimization search task is finished. To normalize the behavior of fireflies, four rules are proposed in the search model.

(i) Every firefly progresses towards brighter fireflies.
(ii) There is always a random movement by the brightest firefly in the group.
(iii) The objective function helps to assess the light intensity of a firefly.
(iv) Irrespective of gender, every firefly can be attracted to other fireflies.

The distance between two fireflies a and b at $c_{i}$ and $c_{j}$ is computed with the help of the cartesian distance. The dimension is specified by D and $c_{as}$ is the $s^{th}$ dimension of the $a^{th}$ firefly and is specified as follows:

(42) $$r_{ab}=\sqrt{\sum_{s=1}^{D}{\left(c_{as}-c_{bs}\right)}^{2}}$$

For the two various fireflies $c_{a}$ and $c_{b}$$\left(a\neq b\right)$, their computation of attractiveness is conducted as follows:

(43) $$\beta \left(r_{ab}\right)=\beta_{0}e^{-\gamma r_{ab}^{2}}$$

where the light absorption coefficient is represented as γ. The starting attractive value is denoted as $\beta_{0}$ when $r_{ab}=0$. With the help of the following Equation (44), firefly movement of a is attracted to much brighter firefly b and is expressed as follows:

(44) $$c_{as}=c_{as}+\beta_{0}e^{-\gamma r_{ab}^{2}}\left(c_{bs}-s_{as}\right)+\alpha \left(rand-0.5\right)$$

where the random scaling factor is expressed as $\alpha \in \left[0,1\right]$ and $rand\in \left[0,1\right]$ denotes a random number. Assuming that the prediction accuracy would be influenced by the weights $w_{i}$ and biases $b_{i}$. FA is utilized to optimize the weights and biases of WELM so that good stability and improved accuracy are obtained. For FA optimized WELM, the major steps are as follows:

(i) The parameters of FA and the individuals are initialized.
(ii) The fitness value (considered as the objective function) is computed for the fireflies, assuming their highest brightness.
(iii) The firefly space position is updated by Equation (44)
(iv) The optimal values are updated simultaneously.
(v) Iterative computation: When the maximum number of searches is reached, the next step is taken, or else step (ii) of this procedure is repeated.

Thus, the hybridization of weights and biases is conducted in the hybrid FA-WELM model.

### 4.4. Proposed Application of Capuchin Search Algorithm to Optimize Weighted ELM Model

While foraging in the forest, the natural movement patterns are emulated by this nature-inspired meta-heuristic technique [42]. In three different methods, the foraging of the *capuchins* is performed while wandering. The three different techniques are swinging, climbing, and jumping. The population in CSA is made up of the alpha *capuchins,* who serve as the leader, and the remaining *capuchins* are the followers. For the capuchins, the food sources are found by the leader. The remaining capuchins’ whereabouts are updated as they follow their leader. The leaders help to manage all the *capuchins* in the CSA algorithm, and the following foraging movement schemes are carried out by the *capuchins,* such as leaping on trees and riverbanks, climbing the trees up and down, swinging on trees, and randomly moving on the ground. The mathematical model of this algorithm is expressed as follows:

When tree jumping is carried out by the *capuchins*, the leader position in CSA is expressed as follows:

(45) cki=Fk+Pbfvki2sin2θgi<n2;0.1≤ε≤0.2

In the $k^{th}$ dimension, the present position of the leader is indicated by $c_{k}^{i}$. In the $k^{th}$ dimension, the food position is represented by $F_{k}$. A random number created uniformly is represented by ε and it is in the range of [0,1]. The tails of the *capuchins* provide the balance probability $P_{bf}$ and it equals 0.8 in our experiment. The gravitational form is expressed by g and the jumping angle of the leader is specified by θ. Over the iteration course, the parameters that mitigate regularly are represented by τ and $v_{k}^{i}$ denote the velocity of the $i^{th}$ capuchin in the $k^{th}$ dimension.

(46) $$\theta =\frac{3}{2}r$$

where the random number is represented by r in the interval of [0,1].

(47) $$\tau =2e^{-21}{\left(\frac{t}{T}\right)}^{2}$$

where t represents the current iteration values and T specifies the maximum iteration values. In the $k^{th}$ dimension, the velocity of the $i^{th}$ capuchin is represented as follows:

(48) $$v_{k}^{i}=\rho v_{k}^{i}+\tau z_{1}\left(c_{best_{k}}^{i}-c_{k}^{i}\right)+\tau z_{2}\left(F_{k}-c_{k}^{i}\right)r_{2}$$

where the present velocity of the $i^{th}$ *capuchin* is specified by $v_{k}^{i}$. The present position of the $i^{th}$ capuchin is represented by $c_{k}^{i}$. The best position of the $i^{th}$ *capuchin* is represented by $c_{best_{k}}^{i}$. The constants are represented by $z_{1}$ and $z_{2}$ that helps to control some parameters.

The random numbers generated in the range of 0 to 1 are represented by $r_{1}$ and $r_{2}$, respectively. The inertia parameter is denoted by ρ that helps to mitigate the previous velocity effects. With the help of the leaping mechanism, the leader positions are determined while foraging on the riverbanks and are represented as follows:

(49) $$c_{k}^{i}=F_{k}+\frac{P_{e}P_{bf}{\left(v_{k}^{i}\right)}^{2}\sin\left(2\theta \right)}{g}i<n/2;0.2\leq \varepsilon \leq 0.3$$

where the elasticity probability of the movement of capuchin is expressed as $P_{e}$ and is fixed as 0.8 in our experiment. When normal walking is employed, the leader position is expressed as follows when the foraging for food happens in the ground and represented as follows:

(50) $$c_{k}^{i}=c_{k}^{i}+v_{k}^{i}i<n/2;\begin{array}{cc} & 0.4<\varepsilon \end{array}<0.5$$

While swinging on trees, the leader’s position is expressed as follows:

(51) $$\begin{array}{l} c_{k}^{i}=F_{k}+\tau P_{bf}\times \sin\left(2\theta \right)\\ i<n/2;\;0.5<\varepsilon \leq 0.75 \end{array}$$

When climbing trees, the leader’s position is expressed as follows:

(52) $$\begin{array}{l} c_{k}^{i}=F_{k}+\tau P_{bf}\left(v_{k}^{i}-v_{k-1}^{i}\right)\\ i<n/2;\;0.75<\varepsilon \leq 1 \end{array}$$

where the previously measured velocity of the $i^{th}$ capuchin in the $k^{th}$ dimension is expressed as $v_{k-1}^{i}$. During the foraging process, the leaders in CSA are randomly relocated and expressed as follows:

(53) $$c_{k}^{i}=\tau \times \left[lb_{k}+\varepsilon \times \left(ub_{k}-lb_{k}\right)\right]i<n/2;\varepsilon \leq \mathrm{Pr}$$

For the $k^{th}$ dimension, the upper and lower bounds of the search space are expressed as $ub_{k}$ and $lb_{k}$, respectively.

For the leaders, the probability of the random walk search is denoted as Pr with a value of 0.4 assigned in our experiment. Using Equation (54), the followers’ positions are expressed as follows:

(54) $$c_{k}^{i}=\frac{1}{2}\left({\bar{c}}_{k}^{i}+c_{k}^{i-1}\right)\begin{array}{cc} & n/2 \end{array}\leq i\leq n$$

where, at dimension k, the present and previous positions are expressed as $c_{k}^{i}$ and $c_{k}^{i-1}$, respectively. At dimension k, the present leaders’ position is expressed as ${\bar{c}}_{k}^{i}$. Based on a fitness method that is predetermined, the evaluation of every new capuchin position is analyzed. To manage the optimization process, an iterative loop technique is utilized. Here, the creation, management, and updating of all the new positions for the *capuchins* are determined. Unless the maximum number of iterations is reached, the loop is reiterated at every step, and the termination of the convergence process happens.

### 4.5. Proposed Implementation of FA/CSA to the Weighted ELM Hybrid with Adaboost Model

**Figure 4:**
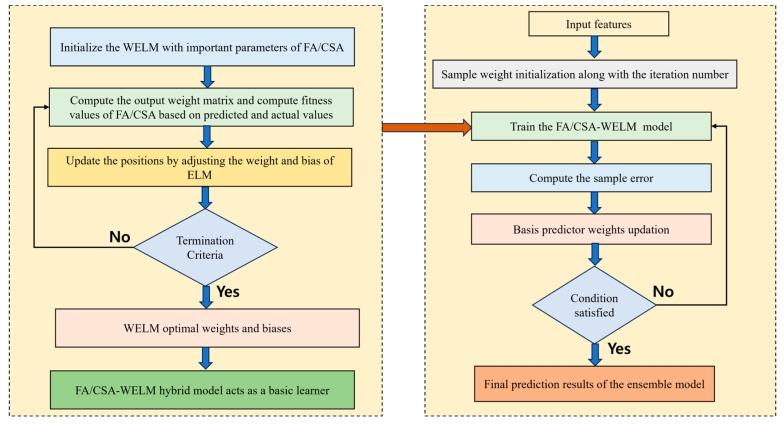
Simplified Illustration of the proposed FA/CSA-WELM- Adaboost hybrid machine learning classifier.

The Adaboost algorithm is highly dependent on the adaptive boosting technique. An implementation of Adaboost for regression issues can be conducted with the help of the Adaboost regressor. The redistribution of weights is performed after the training step is achieved in the Adaboost algorithm. For badly learned data, the weights are increased, and for correctly learned data, the weights are decreased [43]. Therefore, during the training process, huge attention is given to the misfitted data. From every weak regressor, the output is merged and a strong predictor is obtained, which would have few mistakes. To enhance the overall ability of the FA/CSA-WELM model, the Adaboost algorithm is implemented as an ensemble technique. The overall simplified illustration for this hybrid model is given in Figure 4. For the enhancement of the FA/CSA-WELM neural network model, the primary procedure of the Adaboost regressor is as follows:

Input: The feature set $S=\left\{\left(P_{1},q_{1}\right)\left(P_{2},q_{2}\right),\;\ldots ,\;\left(P_{N},q_{N}\right)\right\}$, where the model input features are represented by $P_{i}$ and the respective load data is indicated by $q_{i}$ and FA/CSA-WELM is the weak predictor.

Output: Hybrid Adaboost FA/CSA-WELM model results

The weight vector is initialized first. For the training data, the initialization of the weight distribution is performed as $T_{1}=\left(\frac{1}{N},\frac{1}{N},\;\ldots ,\;\frac{1}{N}\right)$, for K=1,2, …, k.

When the distribution of weights is represented as $T_{k}$, the training of the FA/CSA-WELM model is conducted on the sample data so that the predictor dependent on the classifier is obtained as $h_{k}:S\to Q$.

On the dataset, the prediction error of $h_{k}$ is computed as follows:

(55) $$\varepsilon_{k}^{i}=\left|h_{k}\left(P_{i}\right)-q_{i}\right|/R$$

where $R=\underset{i}{\sup}\left(\left|h_{k}\left(P_{i}\right)-q_{i}\right|\right)$, and the output interval R is in the range of [0,1].

The total error is computed as follows:

(56) $$\varepsilon_{k}=\sum_{i=1}^{n}T_{k}^{i}\varepsilon_{k}^{i}$$

For the current prediction $h_{k}$, the coefficients are computed as follows:

(57) $$c_{k}=\frac{1}{2}\log\left(\frac{1}{\beta_{i}}\right)$$

where,

(58) $$\beta_{i}=\frac{\varepsilon_{k}}{1-\varepsilon_{k}}$$

For the training set, the weight distributions are updated as follows:

(59) $$T_{k}^{i}=\frac{T_{k-1}^{i}.\beta_{K}^{-\varepsilon_{I}}}{Y_{i}}$$

where,

(60) $$Y_{i}=\sum_{i=1}^{N}T_{k}^{i}$$

The connection weights $W=\left(w_{1},w_{2},\;\ldots ,\;w_{k}\right)$ are recorded, and the loop is terminated for the FA/CSA-WELM predictor where $w_{i}=\frac{c_{i}}{\sum_{i=1}^{k}c_{i}}$. Depending on the connection weights, the integration of the trained predictors is performed so that the final strong predictor is obtained as follows:

(61) $$h\left(p\right)=w_{1}h_{1}(p)+w_{2}h_{2}(p)+....+w_{k}h_{k}(p)$$

## 5. Results and Discussion

The publicly available MPSSC dataset was used in the work, and it was presented in the computational paralinguistic challenge of Interspeech 2017 [18]. From three various medical centers, the acquisition of the snoring sounds was performed. The labeling of these sounds was conducted in four classes termed VOTE, where vibration levels of volume are specified by V, oropharyngeal area is denoted by O, tongue is specified by T, and epiglottis is denoted by E, respectively. With a 16 KHz frequency, the pre-processing of the collected sound signals was conducted in 16 bits. In this dataset, there are 828 sounds, and it comprises three important folders: train, development, and test. In this experiment, for training purposes, two of the three categories are utilized, and for testing, the remaining one is utilized. To evaluate our experiments, MATLAB 2020a was utilized on a desktop computer that has a microprocessor of 3.2 GHz, an i7 processor, the Windows 10 operating system, and 32 GB of main memory. For classification using machine learning, a 10-fold cross validation method was utilized. In the previously proposed techniques, UAR (%) was utilized as an evaluation metric, and so for comparison purposes, this metric has exhaustively been concentrated in this work.

As far as the GEO algorithm is concerned, the number of runs is set at 20, the number of iterations is assigned at 200, and the number of search agents is set at 15, respectively. The search domain range is {0,1}, α value is set as 0.8, and β value is set as 0.01 in our experiment. The value of θ is assigned as 0.5, based on Equation (11). As far as the SSA and refined SSA algorithms are concerned, the parameter settings are as follows, λ is assigned as 0.2, the maximum number of iterations are assigned as 200, $w_{\min}$ value is assigned as 0.6 and $w_{\max}$ value is assigned as 0.8, respectively. The range of the lower and upper bounds are assigned as $\left[lp_{k},up_{k}\right]=[0,1]$ and l value is assigned as 0.2 in our experiment. For the hybrid Adaboost WELM model, the number of iterations T and the regularization coefficient λ are quite important. The two important parameters of kernel function μ,γ are also vital in assessing the hybrid model. The values are determined in the range of $\lambda \in \left\{10^{-1},10^{-2},\;\ldots ,\;10^{-10}\right\}$, $T\in \left\{1,2,\;\ldots ,\;20\right\}$, $\mu \in \left\{0,0.1,\;\ldots ,\;0.8\right\}$ and $\gamma \in \left\{10^{0},10^{-1},\;\ldots ,\;10^{-5}\right\}$. When the proposed CSA-WELM-Adaboost hybrid model is implemented, the parameters set for the CSA algorithm are as follows. The number of search agents is assigned as 45, and the number of iterations is set as 200. The number of independent runs is set to 50, α value is set as 0.85 and β value is set as 0.02 in our experiment.

Table 1 shows the performance analysis of time domain features with suitable feature selection techniques and classifiers. Table 2 shows the performance analysis of frequency domain features with suitable feature selection techniques and classifiers. Table 3 shows the performance analysis of DWT domain features with suitable feature selection techniques and classifiers. Table 4 shows the performance analysis of sparse domain features with suitable feature selection techniques and classifiers. Table 5 shows the performance analysis of eigen value domain features with suitable feature selection techniques and classifiers. Table 6 shows the performance analysis of cepstral domain features with suitable feature selection techniques and classifiers. The GEO, SSA, and refined SSA are very new algorithms for comparison’s sake; they have been compared with the standard and old swarm intelligence algorithms like Genetic Algorithm (GA), Ant Colony Optimization (ACO) and Particle Swarm Optimization (PSO) techniques. The machine learning classifiers used here are the Decision Trees (DTs), Naïve Bayesian Classifier (NBC), Random Forest (RF), Logistic Regression (LR), Linear Discriminant Analysis (LDA), SVM with linear kernel, Adaboost, and WELM classifiers.

Table 1 shows that the highest UAR of 70.98% is obtained when the time domain features are selected with the GEO feature selection technique and classified with the proposed FA-WELM-Adaboost classifier. A high UAR of 70.92% is obtained when the time domain features are selected with the GEO feature selection technique and classified with the proposed CSA-WELM-Adaboost classifier.

Table 2 shows that the highest UAR of 70.71% is obtained when the frequency domain features are selected with the refined SSA feature selection technique and classified with the proposed CSA-WELM-Adaboost classifier. A high UAR of 69.88% is obtained when the frequency domain features are selected with the refined SSA feature selection technique and classified with the proposed FA-WELM-Adaboost classifier.

Table 3 shows that the highest UAR of 74.23% is obtained when the DWT domain features are selected with the refined SSA feature selection technique and classified with the proposed FA-WELM-Adaboost classifier. A high UAR of 73.86% is obtained when the DWT domain features are selected with the GEO feature selection technique and classified with the proposed CSA-WELM-Adaboost classifier.

Table 4 shows that the highest UAR of 69.51% is obtained when the sparse domain features are selected with the GEO feature selection technique and classified with the proposed CSA-WELM-Adaboost classifier. A high UAR of 68.90% is obtained when the sparse domain features are selected with the refined SSA feature selection technique and classified with the proposed FA-WELM-Adaboost classifier.

Table 5 shows that the highest UAR of 68.46% is obtained when the Eigen domain features are selected with the GEO feature selection technique and classified with the proposed CSA-WELM-Adaboost classifier. A high UAR of 68.24% is obtained when the eigen domain features are selected with the refined SSA feature selection technique and classified with the proposed FA-WELM-Adaboost classifier.

**Figure 5:**
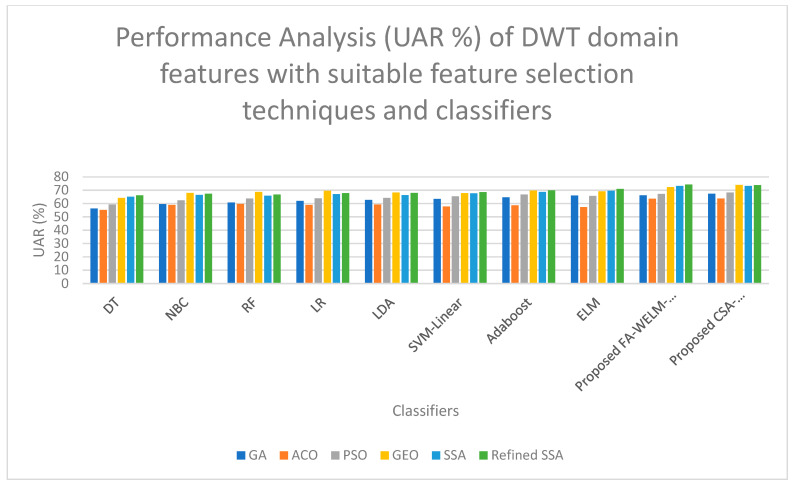
Performance Comparison of classifiers for the DWT features with efficient feature selection schemes.

**Figure 6:**
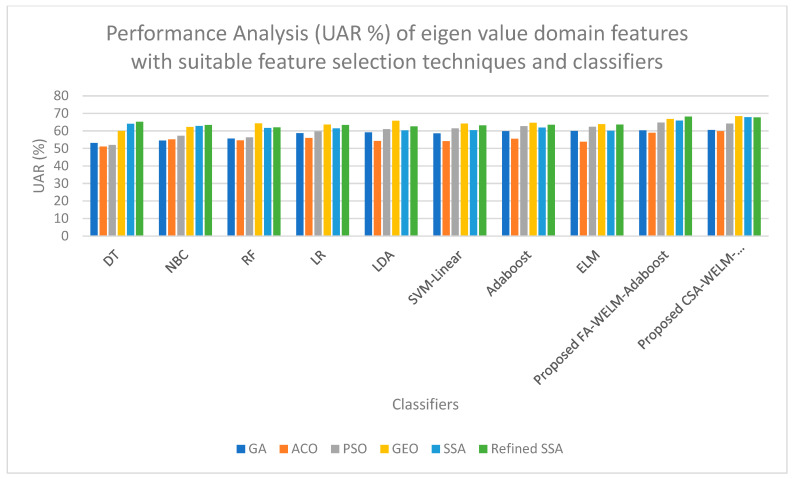
Performance Comparison of classifiers for Eigen value features with efficient feature selection schemes.

Table 6 shows that the highest UAR of 69.86% is obtained when the cepstral domain features are selected with the GEO feature selection technique and classified with the proposed CSA-WELM-Adaboost classifier. A high UAR of 69.81% is obtained when the cepstral domain features are selected with the refined SSA feature selection technique and classified with the proposed FA-WELM-Adaboost classifier. Figure 5 shows the performance comparison of classifiers for the DWT features with efficient feature selection schemes. Figure 6 shows the performance comparison of classifiers for the Eigen value features with efficient feature selection schemes. As DWT features produce the highest UAR and eigen domain features produce the comparatively lower UAR, both of these charts are drawn for comparison purposes. On examining Figure 5, it is evident that the refined SSA feature selection technique with proposed FA-WELM-Adaboost classifiers performs better than the other classifiers, and on examining Figure 6, it is evident that the GEO feature selection technique with proposed CSA-WELM-Adaboost classifiers performs better than the other classifiers.

### Performance Comparison with Previous Works

The obtained results from the experiment are compared with the previous works performed on the same dataset and expressed in Table 7.

On observing Table 7, it is understood that the proposed works produced a high UAR when compared to the previous works. Only one work reported a high UAR of 94.65% in the previous works [22] as the authors have tried a different strategy by employing multiple algorithms and segregating each level independently by using the Leave One Out Cross-Validation (LOOCV) technique. Other than this particular result, the proposed results surpassed all the results of the previous works by obtaining a higher UAR, thereby proving the efficiency and versatility of the proposed works. The main merits of the proposed techniques are as follows. A high UAR is obtained with the implementation of the proposed schemes. Hand crafted features were extracted, selected, and classified with traditional and proposed machine learning classifiers, so the overall computational complexity of the proposed model is lower. The performance evaluation is robust, as is clear from the computational complexity obtained. The overall results reported a lower computational complexity of $O\left(n^{3}\log n\right)$, proving its efficiency and versatility. With the usage of metaheuristic algorithms, there is an added advantage to enhancing the success of the proposed models, and finally, the performance analysis was thoroughly performed and reported in this work.

## 6. Conclusions and Future Work

One of the main reasons for sleep disruption is snoring, which occurs when the airways are partially blocked or restricted when breathing occurs. Snoring is also a serious symptom of many sleep-related disorders, like sleep apnea, cerebral diseases, etc. To assess the severity of snoring, PSG is widely used, but it requires significant cost and time. Classification of snoring is difficult as the snoring period varies from individual to individual depending on the period, length, and frequency of the snoring episodes. Therefore, automated snoring classification algorithms are quite essential, and in this work, six feature extraction domains with three efficient feature selection techniques and ten machine learning classifiers are successfully utilized. The best results are obtained when the DWT features with the refined SSA feature selection technique and FA-WELM-Adaboost hybrid classifier are used, reporting an UAR of 74.23%, while the least UAR is obtained when the cepstral features are utilized with the ACO algorithm and classified with the DT classifier. Future works include the possible usage of other optimization algorithms coupled with more efficient machine learning techniques so that the UAR could be greatly improved. The proposed scheme or strategy could be used for other acoustic genre classification works too. Future works aim to explore a lot of data augmentation types and plans to incorporate their suitability for handling various snoring types, thereby assessing their effectiveness in detecting the snoring in different environments. Future works also aim to deal with very large, noisy datasets so that deep learning can be applied efficiently. Future work also aims to develop a telemedicine-based remote health care monitoring system for managing snoring sound analysis and classification.

## Figures and Tables

**Table 1:** Performance Analysis (UAR %) of Time domain features with suitable feature selection techniques and classifiers.

Classifiers	GA	ACO	PSO	GEO	SSA	Refined SSA
DT	55.65	52.34	58.45	59.55	59.33	61.34
NBC	58.34	52.34	59.67	62.67	63.45	64.56
RF	59.35	57.55	62.87	63.89	63.67	65.77
LR	56.35	52.67	59.32	62.43	61.86	62.44
LDA	55.36	51.89	58.44	64.11	65.23	66.21
SVM-Linear	60.21	56.82	62.56	64.23	64.21	65.33
Adaboost	61.23	55.34	63.89	66.45	65.34	65.46
ELM	62.22	59.11	63.75	67.67	64.67	65.89
Proposed FA-WELM-Adaboost	64.12	58.25	64.21	70.98	68.53	69.75
Proposed CSA-WELM-Adaboost	64.01	57.87	65.87	70.92	69.98	70.24

**Table 2:** Performance Analysis (UAR %) of Frequency domain features with suitable feature selection techniques and classifiers.

Classifiers	GA	ACO	PSO	GEO	SSA	Refined SSA
DT	52.56	55.98	59.09	60.98	61.09	62.01
NBC	57.34	54.76	60.98	63.65	62.67	63.20
RF	57.56	59.78	61.76	64.46	63.87	64.33
LR	55.78	54.98	57.56	61.78	62.33	63.45
LDA	57.92	54.23	59.78	62.93	64.21	66.78
SVM-Linear	62.12	52.46	63.73	60.22	62.45	64.64
Adaboost	62.34	53.76	61.24	64.35	64.67	65.23
ELM	61.56	58.55	60.58	64.68	62.87	64.45
Proposed FA-WELM-Adaboost	62.76	59.41	65.31	69.54	67.54	69.88
Proposed CSA-WELM-Adaboost	63.18	59.02	64.22	70.11	68.17	70.71

**Table 3:** Performance Analysis (UAR %) of DWT domain features with suitable feature selection techniques and classifiers.

Classifiers	GA	ACO	PSO	GEO	SSA	Refined SSA
DT	56.23	55.09	59.22	64.09	65.09	66.11
NBC	59.45	58.98	62.34	67.89	66.46	67.23
RF	60.67	59.68	63.67	68.68	65.78	66.65
LR	61.87	58.94	63.87	69.54	66.98	67.78
LDA	62.64	59.25	64.21	68.24	66.23	67.96
SVM-Linear	63.34	57.78	65.34	67.78	67.57	68.45
Adaboost	64.58	58.61	66.67	69.65	68.66	69.79
ELM	65.98	57.23	65.63	69.11	69.52	70.83
Proposed FA-WELM-Adaboost	66.11	63.55	67.22	72.26	73.11	74.23
Proposed CSA-WELM-Adaboost	67.25	63.78	68.16	73.86	73.09	73.68

**Table 4:** Performance Analysis (UAR %) of sparse domain features with suitable feature selection techniques and classifiers.

Classifiers	GA	ACO	PSO	GEO	SSA	Refined SSA
DT	54.45	52.09	57.12	63.05	64.11	65.03
NBC	57.67	57.87	60.39	65.66	63.23	64.46
RF	58.98	56.78	61.78	66.89	62.78	63.73
LR	60.22	57.89	62.22	66.87	62.43	62.57
LDA	61.12	56.43	63.45	66.22	60.46	62.78
SVM-Linear	60.46	55.35	62.78	65.46	61.86	64.22
Adaboost	62.87	57.78	64.97	67.82	63.36	65.38
ELM	64.21	54.32	62.11	64.46	62.98	66.85
Proposed FA-WELM-Adaboost	64.34	62.11	64.25	68.78	67.25	68.90
Proposed CSA-WELM-Adaboost	62.77	61.57	65.89	69.51	68.75	69.46

**Table 5:** Performance Analysis (UAR %) of eigen value domain features with suitable feature selection techniques and classifiers.

Classifiers	GA	ACO	PSO	GEO	SSA	Refined SSA
DT	53.12	51.09	52.01	60.02	64.09	65.23
NBC	54.45	55.23	57.22	62.22	62.87	63.45
RF	55.68	54.67	56.34	64.34	61.74	62.06
LR	58.75	55.98	59.78	63.67	61.52	63.45
LDA	59.13	54.22	60.97	65.84	60.33	62.62
SVM-Linear	58.56	54.12	61.51	64.25	60.47	63.14
Adaboost	59.88	55.56	62.74	64.67	61.89	63.52
ELM	59.98	53.78	62.35	63.89	60.09	63.69
Proposed FA-WELM-Adaboost	60.31	58.99	64.72	66.83	65.89	68.24
Proposed CSA-WELM-Adaboost	60.56	59.87	64.25	68.46	67.88	67.78

**Table 6:** Performance Analysis (UAR %) of Cepstral domain features with suitable feature selection techniques and classifiers.

Classifiers	GA	ACO	PSO	GEO	SSA	Refined SSA
DT	54.09	50.23	53.04	62.99	61.98	63.11
NBC	55.88	56.56	58.33	63.83	62.57	64.26
RF	56.98	53.74	55.56	63.23	62.34	64.86
LR	59.34	56.35	58.78	62.44	63.79	65.32
LDA	61.56	53.66	61.77	64.67	62.81	65.47
SVM-Linear	59.77	56.82	62.62	66.65	63.25	66.87
Adaboost	58.89	54.14	61.11	63.16	62.79	66.33
ELM	60.19	54.36	61.23	65.55	62.22	65.45
Proposed FA-WELM-Adaboost	61.02	59.85	65.45	68.47	65.56	69.81
Proposed CSA-WELM-Adaboost	62.79	60.47	65.67	69.86	66.87	68.19

**Table 7:** Comparison of the obtained results with previous work conducted on the MPSCC dataset.

Reference	Technique Used	UAR (%)
[12]	MFCC with SVM and ELM	49.38
[13]	CNN and AlexNet with VGG19	67.0
[14]	Deep CNN	72.6
[15]	SVM	49.58
[16]	GMM with DNN	69.71
[17]	LBP and HOG	66.5
[18]	MFCC with SVM	55.8
[19]	Dual Convolution and GRU	63.8
[20]	Wavelet Features	69.4
[21]	Conditional GAN	67.4
[22]	Local dual octal pattern with multilevel DWT, RFINCA and KNN	94.65
Proposed Works	DWT features with refined SSA technique and FA-WELM-Adaboost hybrid classifier	74.23
	DWT features with GEO and CSA-WELM-Adaboost hybrid classifier	73.86
	Time domain features with GEO and FA-WELM-Adaboost hybrid classifier	70.98
	Time domain features with GEO and CSA-WELM-Adaboost hybrid classifier	70.92
	Frequency domain features with refined SSA and CSA-WELM-Adaboost hybrid classifier	70.71

## Data Availability

The publicly available dataset can be referred from “C. Janott, M. Schmitt, Y. Zhang, K. Qian, V. Pandit, Z. Zhang, et al. Snoring classified: the Munich-Passau snore sound corpus Comput. Biol. Med., 94 (2018), pp. 106–118”.
